# Quality of life of adult vitiligo patients using camouflage: A survey in a Chinese vitiligo community

**DOI:** 10.1371/journal.pone.0210581

**Published:** 2019-01-24

**Authors:** Dian Chen, HsiaoHan Tuan, Eray Yihui Zhou, DeHua Liu, Yi Zhao

**Affiliations:** Department of Dermatology, Beijing Tsinghua Changgung Hospital, Tsinghua University, Beijing, China; San Gallicano Dermatologic Institute, ITALY

## Abstract

**Background:**

Vitiligo is an acquired depigmented skin disease resulting in white macules, which may significantly impair the quality of life (QoL) of the patients.

**Objective:**

To estimate the QoL in Chinese vitiligo patients using camouflage with a more detailed description, and to identify the possible risk factors related to poor QoL.

**Methods:**

An online survey was conducted in vitiligo patients using camouflage from a vitiligo community. Survey questions included demographic, clinical information, dermatology- and vitiligo-specific QoL questionnaires. Multivariate logistic analysis was performed to identify risk factors that related to poor QoL.

**Results:**

In total, 884 respondents were included in the analyses, of which 413 (46.7%) were male. The score of DLQI was 5.83±5.75 (mean± SD). Age, gender, marriage status, occupational status, anogenital involvement, patient-perceived severity (presented by VAS score), symptoms as itching, pain, sunburn and koebner phenomenon, total cost of treatment and degree of satisfaction in camouflage therapy were independently associated with DLQI score (p<0.05).

**Conclusion:**

Vitiligo has considerable impact on QoL of affected patients in Chinese population even when they were using camouflage. Camouflage might be helpful to improve QoL of the patients.

## Introduction

Vitiligo is an acquired chronic depigmenting disorder of the skin, predominantly asymptomatic, manifested by circumscribed depigmented macules and patches due to the disappearance of melanocytes from the epidermis. Vitiligo was recognized in ancient times and is still confused with leprosy in some countries[1]. Numerous studies have revealed that vitiligo has a major effect on the patients’ quality of life (QoL) and negatively affects sexual relations[2].

Cosmetic camouflage is an alternative and complementary option to the traditional standard treatment options, since the effectiveness of the latter are often disappointing with only partial results, and the treatment may last for months or years, before re-pigmentation occur. Preliminary studies have reported that camouflage for patients with vitiligo could improve their quality of life[3,4]. However, all these studies were conducted in small populations, therefore this effect remains to be observed in a larger population. Furthermore, the effect of camouflage on QoL can be significantly influenced by social-psychological factors but has not been studied in Chinese population.

We conducted this study to determine the dermatology-specific QoL in a group of adult vitiligo patients using camouflage in a large Chinese vitiligo community, especially to identify the possible risk factors for poor QoL. We also used a vitiligo-specific QoL questionnaire to illustrate the vitiligo-specific QoL aspects that haven’t been covered by DLQI.

## Methods

An online survey was conducted from September to December 2016 to assess QoL of adult vitiligo patients using camouflage, and to identify risk factors that related to poor QoL.

### Recruitment

An online questionnaire was formulated and sent to 8602 consecutive adult patients (age ≥18 years) with vitiligo, in WeChat groups and QQ groups of a vitiligo community, “Leukoplakia Common Home”. Before taking the survey, participants had to sign the online consent form first. Patients who had been using camouflage for more than 1 month were invited. Patients who were younger than 18 years, and who had not been diagnosed by dermatologists were excluded from the study. Information that could identify individual participants was not collected.

The study protocol was approved by the ethics committee of Beijing Tsinghua Changgung Hospital.

### Measures

#### Demographic and clinical Information

Demographic and clinical information was collected including gender, age, residential location, marital status, fertility status, educational level, employment status, income, duration of the disease, localizations of vitiligo (e.g. face, neck, scalp, upper arms, forearms, hands, thighs, legs, feet, chest, upper back, waist, axillae, groins, anogenital area), percentage of body surface area (BSA) affected, symptoms, duration of camouflage therapy, degree of satisfaction in camouflage therapy, other previous treatment, and total cost of treatment, as well as visual analogue scale (VAS) to assess the patient-perceived severity.

#### Quality of life

We used a dermatology-specific QoL questionnaire, Dermatology Life Quality Index (DLQI) and a vitiligo-specific QoL questionnaire, Vitiligo Impact Scale-22 (VIS-22), to assess the impact of vitiligo. The DLQI contains 10 questions related to patients’ symptoms and feelings, daily activities, leisure, work or school, personal relationships, and treatment over the previous 1 week, and each question has four possible answers scored from 0 to 3. The VIS-22 comprises 22 items covering domains of self-confidence, anxiety, depression, marriage, family worries, social interactions, school/college-related, occupation-related, treatment-related and attitude. Each question has four possible answers scored from 0 to 3. The QoL impairment could be classified into 5 levels according to the total score of DLQI. A DLQI score of 0–1 means no effect at all, 2–5 means a small effect, 6–10 moderate effect, 11–20 large effect, and 21–30 extremely large effect on patient’s life.

### Statistical analysis

The data was carefully checked for consistency and plausibility to guarantee the quality of data. Normally distributed data were expressed as means±SD, whereas variables with a skewed distribution were reported as median (range). Categorical variables were represented by frequency and percentage. Univariate associations were calculated using Mann-Whitney U test or Kruskal-Wallis test with Bonferroni correction. The outcome variables were the sum score of DLQI. Independent variables were the sociodemographic and clinical characteristics. The independent variables that were univariately associated with the outcome variables (P <0.10) were entered into a stepwise multivariate logistic regression models with dichotomized DLQI (6–30 vs. 0–5) as dependent variable. A value of P < 0.05 was considered significant. All calculations were performed with the statistical package Stata 12.0 (Stata Corp., College Station, Tex.).

## Results

**Fig 1:**
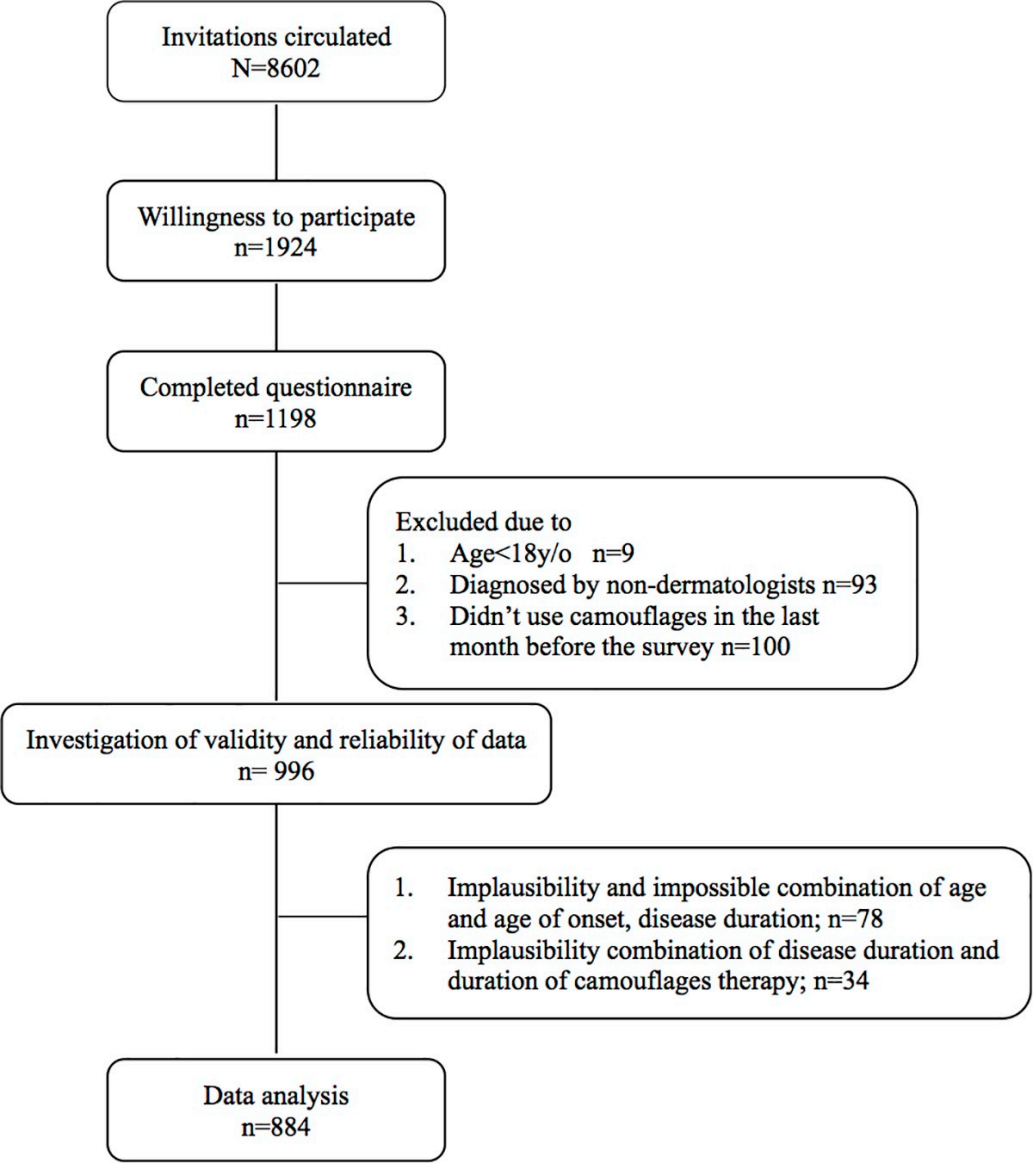
Flow diagram of patient enrollment, exclusion, data quality control and data analysis. (Online completion ensured that there were no missing data in completed questionnaires).

One thousand nine hundred and twenty four patients had shown a willingness to participate in this study. Of whom 1198 completed the questionnaires. However, 314 were excluded after plausibility checking (Fig 1). Therefore, 884 respondents were included in the final analyses.

### Demographic and clinical characteristics of respondents

The median age of these 884 respondents was 36 years (range 18–83); 413 (46.7%) were male. The visible body areas, such as face, scalp, neck, hands, were affected in most of the patients (n = 829, 93.8%). All but one patient had received previous treatment. The median duration of camouflage therapy was 50 months (range 1–216). The demographic and clinical characteristics were presented in Table 1.

**Table 1:** Demographic and clinical characteristics of the study group.

	Study population (n) N = 854	Percent (%)
Gender		
Male	413	46.7
Female	471	53.3
Age, y	38.88 ± 13.10	36 (18–83)^a^
18–29	264	29.9
30–49	421	47.6
50–69	183	20.7
≥ 70	16	1.8
Marital status		
Single, without a committed relationship	101	11.4
Single, in a committed relationship	71	8.0
Married	686	77.6
Divorced	19	2.2
Widowed	7	0.8
Fertility status		
No children	222	25.1
Have child/children	662	74.9
Residential location		
Urban	754	85.3
Rural	130	14.7
Educational level		
Less than high school	132	14.9
High school diploma or equivalent	274	31.0
College graduate	437	49.4
Postgraduate	41	4.6
Employment status		
Employed	662	74.9
Unemployed and Seeking Work	81	9.2
Students	24	2.7
Retire	117	13.2
Onset age, y	23.41 ± 13.03	21 (1–70)
<20	391	44.2
20–29	244	27.6
30–40	127	14.4
≥ 40	122	13.8
Disease duration, y	15.47 ± 10.38	14 (0–55)
<5	119	13.5
5–10	153	17.3
10–20	358	40.5
≥ 20	254	28.7
Number of affected body sites	4.87 ± 3.83	4 (1–15)
Visible body areas affected^b^	829	93.8
Localization^c^		
Face	561	63.5
Neck	389	44.0
Scalp	194	22.0
Upper arms	233	26.4
Forearms	246	27.8
Hands	604	68.3
Thighs	252	28.5
Legs	254	28.7
Feet	340	38.5
Chest	243	27.5
Upper back	205	23.2
Waist	314	35.5
Axillae	170	19.2
Groins	134	15.2
Anogenital	163	18.4
Extension (%BSA)		
<1%	355	40.2
1–3%	207	23.4
3–10%	162	18.3
>10%	160	18.1
Symptoms		
Pruritus	100	11.3
Pain	12	1.4
Sunburn	210	23.8
Koebner phenomenon	300	33.9
Asymptomatic	455	51.5
VAS score of severity	4.77 ± 2.79	4.9 (0–10)
Total cost of treatment		
<10,000 RMB	278	31.5
10,000–50,000 RMB	398	45.0
>50,000–10,000 RMB	208	23.5
Duration of camouflage therapy, m	58.1 ± 44.3	50 (1–216)
Degree of satisfaction in camouflage use		
Not at all	18	2.0
A little	239	27.0
A lot	545	61.7
Very much	82	9.3

^a^ All quantitative data were presented as mean ± SD, median (range).

^b^ The visible body areas were defined as face, scalp, neck, and hands.

^c^ Multiple-choice questions with more than one answer; the sum of percentage >100%.

### Quality of life

**Fig 2:**
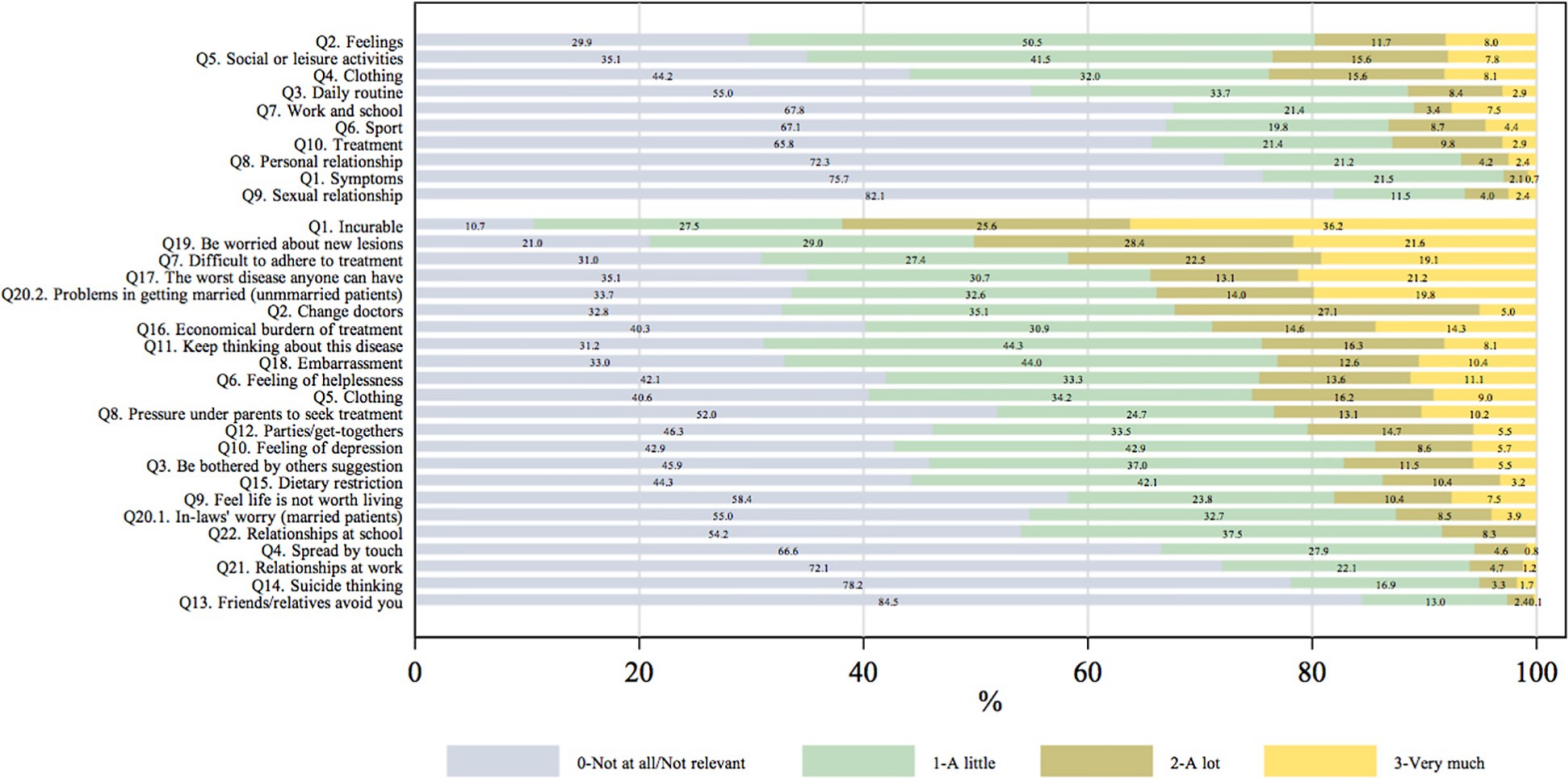
Frequency of the answers to each item of the DLQI and VIS-22 questionnaires, ordered by mean scores of each item.

The DLQI score (mean± SD) was 5.83±5.75 (range 0–30), and 228 (25.8%) patients reported DLQI scores between 0–1, which mean no effect at all; 294 (33.3%) reported small effects; 198 (22.4%), moderate effects; 164 (18.5%), large to extremely large effects. The mean± SD score for the six domains of DLQI were 1.47±1.52 for daily activities, 1.47±1.53 for leisure, 1.25±1.14 for symptoms and feelings, 0.63±1.22 for personal relationship, 0.51±0.88 for work and school, and 0.49±0.79 for treatment. The frequency of the answers to each item of the DLQI and VIS-22 were shown in Fig 2.

### Factors associated with quality of life

#### Univariate associations

The dermatology-specific QoL was more impaired in female patients with vitiligo, and those who were younger than 30y/o, and who live in rural areas had significantly higher scores on DLQI.

The involvement of any part of the body except for face and upper arms, as well as greater percentages of BSA affected were associated with significantly higher scores of DLQI. Patients with symptoms, such as pruritus, pain, koebner phenomenon, and sunburn, had significantly higher DLQI scores compared with those without.

Marriage, fertility and occupational status, as well as total cost of treatment, and the degree of satisfaction in camouflage therapy also had influences on DLQI (S1 Table).

#### Multivariate associations

Multivariate logistic regression analysis revealed that gender, age, marriage status, occupational status, anogenital involvement, patient-perceived severity (presented by VAS score), symptoms as itching, pain, sunburn and koebner phenomenon, total cost of treatment and degree of satisfaction in camouflage therapy were statistically independently associated with DLQI score (Table 2).

**Table 2:** Multivariate logistic regression to identify sociodemographic and clinical factors related to poor health-related quality of life^a^.

		DLQI	
	OR	95% CI	P
Gender (female vs. male)	1.79	1.28~2.52	0.001
Age (≥30 vs. <30 y/o)	0.45	0.30~0.66	<0.001
Marriage (Single, without a committed relationship vs. married)	2.38	1.38~4.11	0.002
Occupational status (Employed vs. retire)	1.70	1.03~2.81	0.038
Occupational status (Unemployed and Seeking Work vs. retire)	2.11	1.04~4.29	0.038
Anogenital involvement	1.92	1.22~3.01	0.004
Hands involvement	1.47	1.00~2.16	0.052
Itching	2.54	1.51~4.29	<0.001
Pain	12.76	1.25~130.71	0.032
Sunburn	2.80	1.90~4.13	<0.001
Koebner phenomenon	1.83	1.29~2.59	0.001
VAS score of severity	1.20	1.13~1.28	<0.001
Cost of treatment (≥10,000 vs. <10,000 RMB)	1.48	1.02~2.14	0.037
Degree of satisfaction in camouflage use (Very much/ A lot vs. A little/ not at all)	0.49	0.34~0.70	<0.001

Abbreviations: OR, Odds ratio; CI, Confidence interval

^a^ A poor QoL was defined as DLQI scores between 6–30. The logistic regression model fit was tested with the likelihood ratio test (P<0.0001).

## Discussion

### Impact of QoL of vitiligo and the assessment tools

Vitiligo is often perceived as a cosmetic disease, whereas it has a profound and permanent impact on patients’ quality of life (QoL). Numerous studies had revealed the association of stigmatization, distress, depression, anxiety, low self-esteem, social isolation and impact on sexual life with vitiligo[5,6]. DLQI is one of the most widely used dermatology-specific QoL instruments in vitiligo, and the DLQI scores reported by previous studies showed obvious geographical differences, with the highest mean DLQI score of 15.00 reported in a Turkish study[7], followed by two Arab studies (mean score 11.86) [8,9], and ten Indian studies (mean score 9.51)[10–16], while the lowest score was 1.82 in one Italian study[17,18]. Therefore, QoL of vitiligo patients is considerably variable according to different skin phototypes and cultures. However, few studies have reported the impact of vitiligo on Chinese patients’ QoL. Although preliminary studies have shown that camouflage can help improving QoL of the patients[3,4], this have never been studied in Chinese patients.

This is the first study to evaluate the impact of vitiligo on Chinese patients who had been using camouflage. To obtain a more comprehensive profile of QoL, we conducted the study in a large vitiligo community using both dermatology-specific and vitiligo-specific QoL instruments.

Since disease-specific instruments could be more sensitive to disease-specific issues, there were four vitiligo-specific Qol instruments had been developed in recent years, including Vitiligo-specific Quality-of-Life instrument (VitiQoL) in the U.S.[19], Vitiligo Impact Patient Scale (VIPs) in France[20], Vitiligo Life Quality Index (VLQI) in Turkey[7], and Vitiligo Impact Scale-22 (VIS-22) in India[13]; and since China and India shared many similarities in culture and belief, we chose the VIS-22 to estimate the vitiligo-specific Qol in our participants. Compared with DLQI, VIS-22 covered the attitude and anxiety about the disease, as well as family worries. Nevertheless, VIS-22 did not have any questions concerned about the symptoms and sexual life.

### Impairment of QoL found in Chinese vitiligo patients

Eight hundred and eighty four vitiligo patients who had been using camouflage for more than 1 month were available for final analyses. The mean total score of DLQI was 5.83. There were more than one third of the vitiligo patients experiencing a significant impairment of QoL, as 40.9% of the patients reported a moderate to extremely large QoL impairment assessed with DLQI (score 6–30).

The highest DLQI score was found in ‘daily activities’, followed by ‘leisure’ and ‘symptoms and feelings’. The high scores of VIS-22 added more detailed information of QoL in domains of attitude and anxiety about the disease, such as ‘disease incurable’, ‘worried about new lesions’, ‘the worst disease’, and ‘keep thinking about this disease’; and treatment-related burdens, such as ‘difficulties in adhering to treatment’, ‘the amount of money spent on treatment’; then ‘problems in getting married’ and the feelings of ‘embarrassment’ and ‘helpless’, while there were only small impacts on work/school life or personal relationship.

### Risk factors related to QoL

The impact of vitiligo on QoL varies in patients with different clinical and demographic characteristics. In the majority of previous studies, women showed more QoL impairment than men did[18], as did young patients compared to elderly ones[10], married women with vitiligo than singles[21], and patients with involvement on exposed sites than those on unexposed sites[18,22–24]. Our results showed that female patients, patients aged less than 30 y/o, patients who were single and without a committed relationship (compared with who were married), patients who were working or seeking work (compared with who retired), patients with anogenital involvement, patients who experienced pruritus or pain, with symptoms as sunburn or koebner phenomenon, and patients who had spent more than 10,000RMB (vs. <10,000 RMB) on treatment had more impaired QoL. Intriguingly, the presence of vitiligo on visible sites, such as face, neck, scalp was not associated with a poor dermatology-specific QoL, which was inconsistent with the previous studies. Several studies showed that patients had higher scores of QoL when having lesions on visible sites[22,23,25,26]. Nevertheless, there were also several studies did not find any relationship between DLQI score and visibility of lesions[6,10,27]. In our study, since most of participants had visible sites involvement (n = 829, 93.8%), the sample size of those without visible sites involvement may be too small (n = 55, 6.2%) to reach statistical significance. Meanwhile, all of our participants had been using camouflage for more than 1 month. QoL has been shown to be improved in vitiligo patients who had lesions on visible sites after use of camouflage[3,4]. Therefore, we supposed that the effectiveness of hiding the visible vitiligo lesions might have abrogated the impact on the patient’s QoL.

We found that the patients with lesions on hands had poor QoL (p = 0.052). These might be explained by the fact that acral lesions of vitiligo were usually resistant to conventional treatment[28–30] as well as surgical interventions[31], and the effect of camouflage on these visible sites could not sustain for long because of frequent hand-washing.

### Camouflage use and QoL in vitiligo patients

Our result showed a less QoL impairment than that reported by Wang et al.[22], who reported a mean total DLQI score of 8.40 in 101 Chinses vitiligo patients. This may be due to the difference between the target populations of these two studies, as we recruited the patients who had been using camouflage for at least one month, from the community, while in the previous study, dermatology clinic outpatients were enrolled.

There were 67 patients who finished the questionnaire were excluded from our primary analysis, as they used camouflage for less than one month. The mean DLQI score of them was higher than those who had been using camouflage for at least one month (7.22±6.83vs. 5.83±5.75), although the differences between the two groups did not reach statistically significance. One possible reason for the difference might be that continuous camouflage application improved the QoL of vitiligo patients who had lesions on visible sites[3,4]. As another evidence, in our multivariate analysis, the degree of satisfaction after camouflage therapy was positively correlated with QoL of the patients.

Several limitations in our study should be addressed. Firstly, it was an online survey, thus the clinical characteristics such as type of vitiligo, disease activity, and vitiligo extent were unable to be assessed clinically. Secondly, there might be some non-serious respondents participated in the survey, and therefore would increase noise and reduce experimental power of the study[32]. For this reason, we performed a consistency and plausibility check to make sure most of the low-quality data sets submitted by non-serious respondents were excluded from the final analysis[32,33]. Thirdly, although China and India shared many similarities in culture and belief, we found that some items in VIS-22 may not be suitable for our patients, further modification and validation is needed. Furthermore, family history was not assessed and should be considered in future studies.

Despite these limitations, our study demonstrated that vitiligo has considerable impact on QoL of affected patients in Chinese population even when they had been using camouflage for at least one month, and camouflage might be helpful to improve QoL of the patients.

## Supporting information

S1 Table Univariate associations of health-related quality of life and sociodemographic and clinical variables. (DOC)

S1 Questionnaire Questionnaire used in this study in both Chinese and English. (DOCX)

S1 Checklist STROBE Statement—checklist of items that should be included in reports of observational studies. (DOCX)

## References

[1] Ezzedine K, Eleftheriadou V, Whitton M, van Geel N. Vitiligo. Lancet. Elsevier; 2015;386: 74–84. 10.1016/S0140-6736(14)60763-7 25596811

[2] Silverberg JI, Silverberg NB. Association Between Vitiligo Extent and Distribution and Quality-of-Life Impairment. JAMA Dermatol. American Medical Association; 2013;149: 159–164. 10.1001/jamadermatol.2013.927 23560296

[3] Tanioka M, Yamamoto Y, Kato M, Miyachi Y. Camouflage for patients with vitiligo vulgaris improved their quality of life. J Cosmet Dermatol. 2010;9: 72–75. 10.1111/j.1473-2165.2010.00479.x 20367677

[4] Ongenae K, Dierckxsens L, Brochez L, van Geel N, Naeyaert J-M. Quality of life and stigmatization profile in a cohort of vitiligo patients and effect of the use of camouflage. Dermatology (Basel). Karger Publishers; 2005;210: 279–285. 10.1159/000084751 15942213

[5] Ongenae K, Beelaert L, Geel N, Naeyaert J-M. Psychosocial effects of vitiligo. J Eur Acad Dermatol Venereol. 2006;20: 1–8. 10.1111/j.1468-3083.2005.01369.x 16405601

[6] Ongenae K, van Geel N, De Schepper S, Naeyaert J-M. Effect of vitiligo on self-reported health-related quality of life. Br J Dermatol. Blackwell Science Ltd; 2005;152: 1165–1172. 10.1111/j.1365-2133.2005.06456.x 15948977

[7] Şenol A, Yücelten AD, Ay P. Development of a quality of life scale for vitiligo. Dermatology. 2013;226: 185–190. 10.1159/000348466 23735515

[8] Robaee Al AA. Assessment of quality of life in Saudi patients with vitiligo in a medical school in Qassim province, Saudi Arabia. Saudi Med J. 2007;28: 1414–1417. 17768471

[9] Bin Saif GA, Al-Balbeesi AO, Binshabaib R, Alsaad D, Kwatra SG, Alzolibani AA, et al Quality of life in family members of vitiligo patients: a questionnaire study in Saudi Arabia. Am J Clin Dermatol. Springer International Publishing; 2013;14: 489–495. 10.1007/s40257-013-0037-5 23839260

[10] Parsad D, Pandhi R, Dogra S, Kanwar AJ, Kumar B. Dermatology Life Quality Index score in vitiligo and its impact on the treatment outcome. Br J Dermatol. 2003;148: 373–374. 12588405 10.1046/j.1365-2133.2003.05097_9.x

[11] Ramam M, Mehta M, Sreenivas V, Sharma V, Khandpur S, Krishna G. Vitiligo impact scale: An instrument to assess the psychosocial burden of vitiligo. Indian J Dermatol Venereol Leprol. 2013;79: 205 10.4103/0378-6323.107637 23442459

[12] Mishra N, Rastogi MK, Gahalaut P, Agrawal S. Dermatology Specific Quality of Life in Vitiligo Patients and Its Relation with Various Variables: A Hospital Based Cross-sectional Study. J Clin Diagn Res. 2014;8: YC01–3. 10.7860/JCDR/2014/8248.4508 25121050 PMC4129253

[13] Gupta V, Sreenivas V, Mehta M, Khaitan BK, Ramam M. Measurement properties of the Vitiligo Impact Scale-22 (VIS-22), a vitiligo-specific quality-of-life instrument. Br J Dermatol. 2014;171: 1084–1090. 10.1111/bjd.13093 24805089

[14] Budania A, Parsad D, Kanwar AJ, Dogra S. Comparison between autologous noncultured epidermal cell suspension and suction blister epidermal grafting in stable vitiligo: a randomized study. Br J Dermatol. Blackwell Publishing Ltd; 2012;167: 1295–1301. 10.1111/bjd.12007 22897617

[15] Singh C, Parsad D, Kanwar AJ, Dogra S, Kumar R. Comparison between autologous noncultured extracted hair follicle outer root sheath cell suspension and autologous noncultured epidermal cell suspension in the treatment of stable vitiligo: a randomized study. Br J Dermatol. 2013;169: 287–293. 10.1111/bjd.12325 23517382

[16] Sahni K, Parsad D, Kanwar AJ, Mehta SD. Autologous noncultured melanocyte transplantation for stable vitiligo: can suspending autologous melanocytes in the patients' own serum improve repigmentation and patient satisfaction? Dermatol Surg. 2011;37: 176–182. 10.1111/j.1524-4725.2010.01847.x 21269348

[17] Ingordo V, Cazzaniga S, Gentile C, Iannazzone SS, Cusano F, Naldi L. Dermatology Life Quality Index score in vitiligo patients: a pilot study among young Italian males. G Ital Dermatol Venereol. 2012;147: 83–90. 22370571

[18] Amer AAA, Gao X-H. Quality of life in patients with vitiligo: an analysis of the dermatology life quality index outcome over the past two decades. Int J Dermatol. 2016;55: 608–614. 10.1111/ijd.13198 26749040

[19] Lilly E, Lu PD, Borovicka JH, Victorson D, Kwasny MJ, West DP, et al Development and validation of a vitiligo-specific quality-of-life instrument (VitiQoL). J Am Acad Dermatol. 2013;69: e11–8. 10.1016/j.jaad.2012.01.038 22365883

[20] Salzes C, Abadie S, Seneschal J, Whitton M, Meurant J-M, Jouary T, et al The Vitiligo Impact Patient Scale (VIPs): Development and Validation of a Vitiligo Burden Assessment Tool. J Invest Dermatol. 2015;136: 52–58. 10.1038/jid.2015.398 26763423

[21] Dolatshahi M, Ghazi P, Feizy V, Hemami MR. Life quality assessment among patients with vitiligo: comparison of married and single patients in Iran. Indian J Dermatol Venereol Leprol. 2008;74: 700. 10.4103/0378-6323.45141 19177700

[22] Wang K-Y, Wang K-H, Zhang Z-P. Health-related quality of life and marital quality of vitiligo patients in China. J Eur Acad Dermatol Venereol. 2011;25: 429–435. 10.1111/j.1468-3083.2010.03808.x 20666878

[23] Radtke MA, Schäfer I, Gajur A, Langenbruch A, Augustin M. Willingness-to-pay and quality of life in patients with vitiligo. Br J Dermatol. Blackwell Publishing Ltd; 2009;161: 134–139. 10.1111/j.1365-2133.2009.09091.x 19298268

[24] Wong SM, Baba R. Quality of life among Malaysian patients with vitiligo. Int J Dermatol. 2012;51: 158–61. 10.1111/j.1365-4632.2011.04932.x 22250623

[25] Steele R, Min K, Lo A. Quality of life among Malaysian patients with vitiligo. J Assoc Inf Sci Technol. 2017;63: 1079–1091. 10.1111/j.1365-4632.2011.04932.x

[26] Bae JM, Lee SC, Kim TH, Yeom SD, Shin JH, Lee W-J, et al Factors affecting quality of life in patients with vitiligo: a nationwide study. Br J Dermatol. 2017;65: 473–7. 10.1111/bjd.15560 28391642

[27] Sampogna F, Raskovic D, Guerra L, Pedicelli C, Tabolli S, Leoni L, et al Identification of categories at risk for high quality of life impairment in patients with vitiligo. Br J Dermatol. Blackwell Publishing Ltd; 2008;159: 351–359. 10.1111/j.1365-2133.2008.08678.x 18565189

[28] Bae JM, Jung HM, Hong BY, Lee JH, Choi WJ, Lee JH, et al Phototherapy for Vitiligo. JAMA Dermatol. American Medical Association; 2017;153: 666–9. 10.1001/jamadermatol.2017.0002 28355423 PMC5817459

[29] Speeckaert R, Geel N. Vitiligo: An Update on Pathophysiology and Treatment Options. Am J Clin Dermatol. Springer International Publishing; 2017;: 1–12. 10.1007/s40257-016-0238-9 28577207

[30] Esmat SM, El-Tawdy AM, Hafez GA, Zeid OA, Abdel Halim DM, Saleh MA, et al Acral lesions of vitiligo: why are they resistant to photochemotherapy? J Eur Acad Dermatol Venereol. Blackwell Publishing Ltd; 2011;26: 1097–1104. 10.1111/j.1468-3083.2011.04215.x 21851425

[31] Falabella R. Surgical approaches for stable vitiligo. Dermatol Surg. 2005;31: 1277–1284. 16188179 10.1111/j.1524-4725.2005.31203

[32] Aust F, Diedenhofen B, Ullrich S, Musch J. Seriousness checks are useful to improve data validity in online research. Behav Res Methods. 2013;45: 527–535. 10.3758/s13428-012-0265-2 23055170

[33] Reips UD. How internet-mediated research changes science. Gsbhaifaacil. 2008.

