# Supplementary material for: Quality of life of adult vitiligo patients using camouflage: A survey in a Chinese vitiligo community
Source: PLoS One. 2019 Jan 24;14(1):e0210581. doi: 10.1371/journal.pone.0210581 (PMC6345473; doi:10.1371/journal.pone.0210581)
Supplement: S1 Table — (DOC) [file pone.0210581.s001.doc]

S1 Table. Univariate associations of health-related quality of life and sociodemographic and clinical variables

| **Variable** | **No** | **(%)** | **DLQI (mean±SD)** | **P value*** |
| --- | --- | --- | --- | --- |
| Gender |  |  |  |  |
| Male | 413 | 46.7 | 5.22±5.47 | 0.0008 |
| Female | 471 | 53.3 | 6.36±5.93 |  |
| Age, y |  |  |  |  |
| <30 | 264 | 29.9 | 7.53±6.02 | <0.0001 |
| ≥30 | 620 | 70.1 | 5.10±5.47 |  |
| Marital status |  |  |  |  |
| Single, | 101 | 11.4 | 8.50±6.04 | 0.0001 |
| Single, in a committed relationship | 71 | 8.0 | 6.56±5.90 |  |
| Married | 686 | 77.6 | 5.24±5.44 |  |
| Divorced | 19 | 2.1 | 9.47±8.76 |  |
| Widowed | 7 | 0.8 | 7.29±4.86 |  |
| Fertility status |  |  |  |  |
| No children | 222 | 25.1 | 7.55±6.05 | <0.0001 |
| Have child/children | 662 | 74.9 | 5.25±5.53 |  |
| Residential location |  |  |  |  |
| Urban | 754 | 85.3 | 5.60±5.60 | 0.0012 |
| Rural | 130 | 14.7 | 7.28±6.34 |  |
| Educational level |  |  |  |  |
| Less than high school | 132 | 14.9 | 6.48±6.25 | 0.0001 |
| High school diploma or equivalent | 274 | 31.0 | 4.88±5.75 |  |
| College graduate | 437 | 49.4 | 6.30±5.67 |  |
| Postgraduate | 41 | 4.6 | 5.05±3.79 |  |
| Occupational status |  |  |  |  |
| Employed | 662 | 74.9 | 5.84±5.55 | 0.0001 |
| Unemployed and Seeking Work | 81 | 9.2 | 8.90±7.73 |  |
| Students | 24 | 2.7 | 8.88±5.33 |  |
| Retire | 117 | 13.2 | 3.00±3.50 |  |
| Disease Duration, y |  |  |  |  |
| <5 | 119 | 13.5 | 5.71±5.67 | 0.0955 |
| 5-10 | 153 | 17.3 | 6.86±0.32 |  |
| 10-20 | 358 | 40.5 | 5.82±5.79 |  |
| ≥20 | 254 | 28.7 | 5.29±5.28 |  |
| Localization |  |  |  |  |
| Face | 561 | 63.5 | 5.93±5.87 | 0.5895 |
| Non-face | 323 | 36.5 | 5.64±5.52 |  |
| Neck | 389 | 44.0 | 6.77±5.93 | <0.0001 |
| Non-neck | 495 | 56.0 | 5.09±5.50 |  |
| Scalp | 194 | 21.9 | 8.01±6.36 | <0.0001 |
| Non-scalp | 690 | 78.1 | 5.22±5.41 |  |
| Upper arms | 233 | 26.4 | 6.89±6.57 | 0.0103 |
| Non-upper-arm | 651 | 73.6 | 5.45±5.38 |  |
| Forearms | 246 | 27.8 | 7.26±6.58 | <0.0001 |
| Non-forearm | 638 | 72.2 | 5.28±5.29 |  |
| Hands | 604 | 68.3 | 6.21±5.88 | 0.001 |
| Non-hand | 280 | 31.7 | 5.00±5.35 |  |
| Thighs | 252 | 28.5 | 7.39±6.65 | <0.0001 |
| Non-thigh | 632 | 71.5 | 5.21±5.22 |  |
| Legs | 254 | 28.7 | 7.05±6.13 | <0.0001 |
| Non-leg | 630 | 71.3 | 5.34±5.51 |  |
| Feet | 340 | 38.5 | 6.87±6.09 | <0.0001 |
| Non-feet | 544 | 61.5 | 5.18±5.42 |  |
| Chest | 243 | 27.5 | 7.92±6.63 | <0.0001 |
| Non-chest | 641 | 72.5 | 5.04±5.16 |  |
| Upper back | 205 | 23.2 | 7.59±6.39 | <0.0001 |
| Non-upper-back | 679 | 76.8 | 5.30±5.43 |  |
| Waist | 314 | 35.5 | 7.20±6.31 | <0.0001 |
| Non-waist | 570 | 64.5 | 5.08±5.26 |  |
| Axillae | 170 | 19.2 | 8.32±6.34 | <0.0001 |
| Non-axillae | 714 | 80.8 | 5.24±5.43 |  |
| Groins | 134 | 15.2 | 9.13±6.48 | <0.0001 |
| Non-groin | 750 | 84.8 | 5.24±5.40 |  |
| Anogenital | 163 | 18.4 | 9.58±6.85 | <0.0001 |
| Non-anogenital | 721 | 81.6 | 4.98±5.10 |  |
| Extension (%BSA) |  |  |  |  |
| <3% | 562 | 63.6 | 4.99±5.13 | 0.0001 |
| ≥3% | 322 | 36.4 | 7.30±6.44 |  |
| Symptoms |  |  |  |  |
| Pruritus present | 100 | 11.3 | 9.58±6.85 | <0.0001 |
| not present | 784 | 88.7 | 5.35±5.41 |  |
| Pain present | 12 | 1.4 | 11.00±6.93 | 0.0017 |
| not present | 872 | 98.6 | 5.76±5.70 |  |
| Sunburn present | 210 | 23.8 | 8.40±6.43 | <0.0001 |
| not present | 674 | 76.2 | 5.03±5.27 |  |
| Koebner phenomenon present | 300 | 33.9 | 8.32±6.69 | <0.0001 |
| not present | 584 | 66.1 | 4.55±4.71 |  |
| Total cost of treatment |  |  |  |  |
| <10,000 RMB | 278 | 31.4 | 4.39±5.18 | 0.0001 |
| 10,000-50,000 RMB | 398 | 45.0 | 5.83±5.53 |  |
| >50,000-10,000 RMB | 208 | 23.5 | 7.75±6.30 |  |
| Camouflage duration, y |  |  |  |  |
| ≤ 1 | 142 | 16.1 | 6.68±5.49 | 0.0106 |
| 1-5 | 361 | 40.8 | 5.94±6.06 |  |
| > 5 | 381 | 43.1 | 5.41±5.50 |  |
| Degree of satisfaction in camouflage use |  |  |  |  |
| Not at all | 18 | 2.0 | 8.44±7.86 | 0.0001 |
| A little | 239 | 27.0 | 8.20±6.34 |  |
| A lot | 545 | 61.7 | 5.04±5.17 |  |
| Very much | 82 | 9.3 | 3.57±4.67 |  |

*P values indicate comparisons between subgroups of each variable. Because of multiple comparisons, a Bonferroni correction was performed, resulting an adjusted alpha of 0.003 (0.1/31).
