# Supplementary material for: Quality of life of adult vitiligo patients using camouflage: A survey in a Chinese vitiligo community
Source: PLoS One. 2019 Jan 24;14(1):e0210581. doi: 10.1371/journal.pone.0210581 (PMC6345473; doi:10.1371/journal.pone.0210581)
Supplement: S1 Questionnaire — (DOCX) [file pone.0210581.s002.docx]

**Questionnaire (Chinese Version)**

**一般情况调查表**

| 1. | 性别 | 男 □ 女 □ |  |  |  |
| --- | --- | --- | --- | --- | --- |
| 2. | 出生日期 | 年 月 日 | | | |
| 3. | 民族 |  |  |  |  |
| 4. | 常住地 | 省 |  |  | 城市□ 农村□ |
| 5. | 婚姻状况 |  |  |  |  |
| 6. | 未婚（无固定对象）□ 未婚（有固定对象）□ 已婚□ 离异□  丧偶□  生育情况  子女 有□ 无□ | | | | |
| 7. | 文化程度 | | | | |
|  | 文盲□ 小学□ 初中□ 高中（或中专）□ 大学（或大专）□  研究生及以上□ | | | | |
| 8. | 就业状态 | | | | |
|  | 在职□ 失业或待业□ 学生□ 退休□ | | | | |
| 9. | 收入水平 | | | | |
|  | <1000元/月□ 1000-3000元/月□ 3000-5000元／月□  5000-1万元／月□ 1-3万元／月□ >3万元／月□ | | | | |

**皮肤生活质量指数问卷**

这份问卷的目的是衡量***上周内***您的皮肤问题对您的生活造成了多大的影响，请在每个问题后选择一项打“√”

1、上周内，您的皮肤感到痒、触痛、疼痛、刺痛了吗；

□非常多 □许多 □一点 □完全没有

2、上周内，由于您的皮肤问题，您感到尴尬或自卑吗？

□非常多 □许多 □一点 □完全没有

3、上周内，因为皮肤问题，对您购物、做家务、整理庭院影响程度如何？

□非常多 □许多 □一点 □完全没有 □无关

4、上周内，皮肤问题对您穿衣服影响程度如何？

□非常多 □许多 □一点 □完全没有 □无关

5、上周内，皮肤问题对您的社交或休闲生活有多大的影响？

□非常多 □许多 □一点 □完全没有 □无关

6、上周内，皮肤问题对您运动有多大妨碍？

□非常多 □许多 □一点 □完全没有 □无关

7、上周内，皮肤问题是否让您无法上班或学习？

□是 □不是 □无关

如果选择“不是”，那么上周内您的皮肤问题对工作或学习有多大影响呢？

□许多 □一点 □完全没有

8、上周内，皮肤问题妨碍了您和爱人、亲密的朋友、亲戚间的交往了吗？

□非常多 □许多 □一点 □完全没有 □无关

9、上周内，皮肤问题给您的性生活造成了多大影响？

□非常多 □许多 □一点 □完全没有 □无关

10、上周内，由于治疗您皮肤的毛病，给您造成了多少麻烦，如把家里弄得一团糟或占用了您很多时间？

□非常多 □许多 □一点 □完全没有 □无关

请您检查您是否已回答所有问题 谢谢合作

© AY Finlay, GK Khan. April 1992. This must not be copied without the permission of authors

## 白癜风影响量表（VIS-22）

| 本量表是为了评量白癜风对您生活的影响。请仔细阅读，并根据您的理解，回答以下问题。 | |
| --- | --- |
| 1. | 您是否认为这个病不能治愈吗? |
|  | □_0_完全不这么认为 □_1_有一点 □_2_ 比较肯定 □_3_ 肯定 |
| 2. | 您是否更换医生？ |
|  | □_0_完全没有 □_1_偶尔 □_2_ 经常 □_3_ 频繁 |
| 3. | 其他人对本病的建议是否会让您烦恼？ |
|  | □_0_完全没有 □_1_有一些 □_2_ 较多 □_3_ 非常多 |
| 4. | 其他人是否觉您的皮肤病会通过接触传染？ |
|  | □_0_完全没有 □_1_有一些 □_2_ 许多 □_3_ 非常多 |
| 5. | 您在选择着装时是否会有困难？ |
|  | □_0_完全没有 □_1_偶尔 □_2_ 经常 □_3_ 频繁 |
| 6. | 是否会有无助感？ |
|  | □_0_完全没有 □_1_有一些 □_2_ 许多 □_3_ 非常多 |
| 7. | 您对坚持治疗感到困难吗? |
|  | □_0_完全没有 □_1_有一些 □_2_ 许多 □_3_ 非常多 |
| 8. | 您的父母是否一直要求您寻求治疗？ |
|  | □_0_完全没有 □_1_偶尔 □_2_ 经常 □_3_ 频繁 |
| 9. | 您是否因为这个病感到痛不欲生？ |
|  | □_0_完全没有 □_1_偶尔 □_2_ 经常 □_3_ 频繁 |
| 10. | 您是否感觉抑郁？ |
|  | □_0_完全没有 □_1_有一些 □_2_ 许多 □_3_ 非常多 |
| 11. | 您会否一直在脑海里琢磨着这个病？ |
|  | □_0_完全没有 □_1_有一些 □_2_ 许多 □_3_ 非常多 |
| 12. | 您是否减少或停止参加派对或聚会？ |
|  | □_0_完全没有 □_1_偶尔 □_2_ 经常 □_3_ 频繁 |
| 13. | 您的朋友或亲戚是否会避开您？ |
|  | □_0_完全没有 □_1_偶尔 □_2_ 经常 □_3_ 频繁 |
| 14. | 您是否曾因这个病有过轻生的念头？ |
|  | □_0_完全没有 □_1_偶尔 □_2_ 经常 □_3_ 频繁 |
| 15. | 您是否会“忌口”？ |
|  | □_0_完全没有 □_1_偶尔 □_2_ 经常 □_3_ 频繁 |
| 16. | 治疗上的花费是否会困扰您？ |
|  | □_0_完全没有 □_1_有一些 □_2_ 许多 □_3_ 非常多 |
| 17. | 您是否觉得这个病是世界上最糟糕的疾病？ |
|  | □_0_完全没有 □_1_偶尔 □_2_ 经常 □_3_ 频繁 |
| 18. | 在与其他人见面时您是否会觉得尴尬？ |
|  | □_0_完全没有 □_1_偶尔 □_2_ 经常 □_3_ 频繁 |
| 19. | 如果出现了新的白斑，您的担心程度会是？ |
|  | □_0_完全不 □_1_ 有一点 □_2_ 比较严重 □_3_ 十分严重 |
|  | 如果您已婚，请回答以下问题 |
| 20. | 您的爱人是否会担心您的白斑？ |
|  | □_0_完全不 □_1_ 有一点 □_2_ 比较严重 □_3_ 十分严重 |
|  | 若果您未婚，请回答以下问题 |
| 20. | 您正在面临结婚的难题吗? |
|  | □_0_完全没有 □_1_ 有一点 □_2_ 比较严重 □_3_ 十分严重 |
|  | 如果您已参加工作，请回到以下问题 |
| 21. | 您的同事是否因为您的皮肤问题而区别对待您？ |
|  | □_0_完全没有 □_1_偶尔 □_2_ 经常 □_3_ 频繁 |
|  | 如果您在上学，请回答以下问题 |
| 22. | 您的同学是否因为您的皮肤问题而区别对待您？ |
|  | □_0_完全没有 □_1_偶尔 □_2_ 经常 □_3_ 频繁 |

##

## 病史采集表

| 1. | 您初发病时的年龄 | 周岁 | | | |
| --- | --- | --- | --- | --- | --- |
| 2. | 诊断时间 | 年前 | 是否由皮肤科专科医师明确诊断 | | 是□ 否□ |
| 3. | 白斑部位 | （多选） |  | |  |
|  | 面部□ 颈部□ 头皮□ 上臂□ 前臂□ 手□ 大腿□ 小腿□ 足□ 前胸□ 上背□ 腰腹□ 腋窝 腹股沟 肛门生殖器□ | | | | |
| 4. | 白斑面积 | 用您自己的手掌测量（5指并拢），您的白斑面积有多少个手掌大小？（1个手掌相当于1%体表面积，如果您的白斑比较分散，请估计它们加在一起的总面积） | | | |
|  | <1/4手掌□ <1/2手掌□ <1个□ 1-3个□ 3-10个□ 10-25个（1/10-1/4总体表面积）□ 25-50个（1/4-1/2总体表面积）□ >50个（1/2总体表面积）□ | | | | |
| 5. | 症状 | （多选） | |  |  |
|  | 瘙痒□ 疼痛□ 易晒伤□  受到创伤、抓伤、手术切口、日晒伤、针刺、摩擦等损伤伤后，**可诱发新的白斑，或原有白斑扩大**□  无□ | | | | |

## 补充和建议

1. 严重程度（总体来说您觉的您的病情有多严重？）

一点也不严重 极为严重

1. 您是否用过以下治疗，您认为该项治疗的效果？

| 外用药 |  |  |  |  |  |
| --- | --- | --- | --- | --- | --- |
| 外用糖皮质激素（如艾洛松、卤米松、克廷夫）□ | □没用过 | 不满意□ | 有些满意□ | 比较满意□ | 十分满意□ |
| 他克莫司/（普特彼）□ | □没用过 | 不满意□ | 有些满意□ | 比较满意□ | 十分满意□ |
| 吡美莫司（爱宁达）□） | □没用过 | 不满意□ | 有些满意□ | 比较满意□ | 十分满意□ |
| 外用光敏剂（如补骨酯酊）□ | □没用过 | 不满意□ | 有些满意□ | 比较满意□ | 十分满意□ |
| 外用维生素D衍生物（卡泊三醇□ 他卡西醇□） | □没用过 | 不满意□ | 有些满意□ | 比较满意□ | 十分满意□ |
| 遮盖剂□ | □没用过 | 不满意□ | 有些满意□ | 比较满意□ | 十分满意□ |
| 口服或注射 |  |  |  |  |  |
| 口服或注射糖皮质激素（如泼尼松、泼尼松龙、甲泼尼龙（美卓乐）、地塞米松、得宝松（复方被他米松）、曲安西龙等）□ | □没用过 | 不满意□ | 有些满意□ | 比较满意□ | 十分满意□ |
| 硫唑嘌呤□ | □没用过 | 不满意□ | 有些满意□ | 比较满意□ | 十分满意□ |
| 环孢素□ | □没用过 | 不满意□ | 有些满意□ | 比较满意□ | 十分满意□ |
| 甲胺蝶呤□ | □没用过 | 不满意□ | 有些满意□ | 比较满意□ | 十分满意□ |
| 利妥昔单抗□ | □没用过 | 不满意□ | 有些满意□ | 比较满意□ | 十分满意□ |
| 益赛普/类克□ | □没用过 | 不满意□ | 有些满意□ | 比较满意□ | 十分满意□ |
| 口服中药／中成药，如胸腺肽、转移因子、补骨酯注射液□ | □没用过 | 不满意□ | 有些满意□ | 比较满意□ | 十分满意□ |
| 照光： |  |  |  |  |  |
| 窄谱紫外线（UVB）光疗□ | □没用过 | 不满意□ | 有些满意□ | 比较满意□ | 十分满意□ |
| 308nm激光□ | □没用过 | 不满意□ | 有些满意□ | 比较满意□ | 十分满意□ |
| UVA（黑光灯）□ | □没用过 | 不满意□ | 有些满意□ | 比较满意□ | 十分满意□ |
| 手术，如表皮移植、黑素细胞移植□ | □没用过 | 不满意□ | 有些满意□ | 比较满意□ | 十分满意□ |
| 其他：_______________ | □没用过 | 不满意□ | 有些满意□ | 比较满意□ | 十分满意□ |

1. 是否曾使用过遮盖剂？ □是 □否
2. 目前是否仍在使用遮盖剂？ □是 □否
3. 使用遮盖剂的总时间？ 共 年 月
4. 遮盖效果？ 不满意□ 有些满意□ 比较满意□ 十分满意□
5. 迄今在白癜风治疗上的花费总额？

|  | <3000元□ 3000-5000元□ 5000-1万元□ 1-3万元□ 3-5万元□ 5-10万元□ 10-20万元□ >20万元□ |
| --- | --- |

1. 您觉得上述表格是否能反应白癜风所给您带来的影响？

□能 □比较符合 □不太能 □不能

1. 您觉得有哪些是影响明显，而我们没有调查到的？

请补充说明：

1. 若以后有类似的调查，您是否仍愿意参与？

□愿意 □不愿意

**Questionnaire (English Version)**

**Demographic Information**

| 1. | Gender | Male□ Female□ | | | |
| --- | --- | --- | --- | --- | --- |
| 2. | Birthday | YYYY-MM-DD | | | |
| 3. | Ethics |  |  |  |  |
| 4. | Residential location | (Province) |  |  | Urban□ Rural□ |
| 5. | Marital status |  |  |  |  |
| 6. | Single□ Single, in a committed relationship□  Married□ Divorced□ Widowed□  Fertility status  No children□ Have child/children□ | | | | |
| 7. | Education | | | | |
|  | Less than primary school□  Primary school □  Junior middle school□  High school diploma or equivalent □  College graduate□  Postgraduate□ | | | | |
| 8. | employment status | | | | |
|  | Employed□ Unemployed and Seeking Work□  Students□ Retire□ | | | | |
| 9. | income | | | | |
|  | < 1000 RMB/M□ 1000-3000 RMB/M□ 3000-5000 RMB/M□  5000-10000 RMB/M□ 10000-30000 RMB/M□ >30000 RMB/M□ | | | | |

##

## Clinical Information

| 1. | Onset age, y | y/o | | |
| --- | --- | --- | --- | --- |
| 2. | When have you been diagnosis as vitiligo? | years ago | Was the diagnosis made by a dermatologist? | Yes□ No□ |
| 3. | Where are you white patches? (Multi-choice) | | | |
|  | Face□ Neck□ Scalp□ Upper arms□ Forearms□ Hands□ Thighs□ Legs□ Feet□ Chest□ Upper back□ Waist□ Axillae□ Groins□ Anogenital□ | | | |
| 4. | Extension (%BSA) | Use your hand to measure the extension of your white patches (the palmar surface of your hand, including the fingers, represents approximately 1% of your body surface area(BSA)). | | |
|  | <1/4 hand surface area (HSA)□ <1/2 HSA□ <1 HSA□ 1-3 HSA□ 3-10 HSA□ 10-25 HAS (1/10-1/4 BSA)□  25-50 HAS (1/4-1/2 BSA)□ >50 HAS (1/2 BSA)□ | | | |
| 5. | Symptoms (Multi-choice) | | | |
|  | Pruritus□ Pain□ Sunburn□  Koebner phenomenon□  Asymptomatic□ | | | |

## Other information

1. Disease severity (Overall, how severe do you thick of your disease？)

Not at all Extremely severe

1. Have you ever been treated with the treatment options below, and do you satisfy with those treatments?

| Topical therapy |  |  |  |  |  |
| --- | --- | --- | --- | --- | --- |
| Topical corticosteroids□ | □Never | Not satisfied at all □ | A little satisfied□ | Quite satisfied□ | Very satisfied□ |
| Tacrolimus□ | □Never | Not satisfied at all □ | A little satisfied□ | Quite satisfied□ | Very satisfied□ |
| Pimercrilimus□ | □Never | Not satisfied at all □ | A little satisfied□ | Quite satisfied□ | Very satisfied□ |
| Topical photosensitizer□ | □Never | Not satisfied at all □ | A little satisfied□ | Quite satisfied□ | Very satisfied□ |
| Topical vitamin D derivatives  (Calcipotriol□ or  Tacalcitol□） | □Never | Not satisfied at all □ | A little satisfied□ | Quite satisfied□ | Very satisfied□ |
| Camouflage□ | □Never | Not satisfied at all □ | A little satisfied□ | Quite satisfied□ | Very satisfied□ |
| Systemic therapy | □Never | Not satisfied at all □ | A little satisfied□ | Quite satisfied□ | Very satisfied□ |
| Systemic (oral or injectable) corticosteroids (i.e. prednisone, prednisolone, methylprednisolone, dexamethasone, diprosone, triamcinolone, etc)□ | □Never | Not satisfied at all □ | A little satisfied□ | Quite satisfied□ | Very satisfied□ |
| Azathioprine□ | □Never | Not satisfied at all □ | A little satisfied□ | Quite satisfied□ | Very satisfied□ |
| Cyclosporine□ | □Never | Not satisfied at all □ | A little satisfied□ | Quite satisfied□ | Very satisfied□ |
| Methotrexate□ | □Never | Not satisfied at all □ | A little satisfied□ | Quite satisfied□ | Very satisfied□ |
| Rituximab□ | □Never | Not satisfied at all □ | A little satisfied□ | Quite satisfied□ | Very satisfied□ |
| Etanercept / Infliximab □ | □Never | Not satisfied at all □ | A little satisfied□ | Quite satisfied□ | Very satisfied□ |
| Traditional Chinese medicine□ | □Never | Not satisfied at all □ | A little satisfied□ | Quite satisfied□ | Very satisfied□ |
| Phototherapy： | □Never | Not satisfied at all □ | A little satisfied□ | Quite satisfied□ | Very satisfied□ |
| Narrow band UVB□ | □Never | Not satisfied at all □ | A little satisfied□ | Quite satisfied□ | Very satisfied□ |
| 308nm laser□ | □Never | Not satisfied at all □ | A little satisfied□ | Quite satisfied□ | Very satisfied□ |
| UVA□ | □Never | Not satisfied at all □ | A little satisfied□ | Quite satisfied□ | Very satisfied□ |
| Surgery, such as epidermal transplantation and melanocyte transplantation□ | □Never | Not satisfied at all □ | A little satisfied□ | Quite satisfied□ | Very satisfied□ |
| Others：_______________ | □Never | Not satisfied at all □ | A little satisfied□ | Quite satisfied□ | Very satisfied□ |

1. Have you ever used camouflage？ □Yes □No
2. Are you still using camouflage？ □Yes □No
3. How long have you been using camouflage？ years and months
4. Are you satisfied with it？ Not at all□ A little□ A lot□ Very much□
5. How much have you ever spend on the treatment of vitiligo?

|  | <3000 RMB□ 3000-5000 RMB□ 5000-10000 RMB□ 10000-30000 RMB□ 30000-50000 RMB□ 50000-100000RMB□ 100000-200000 RMB□ >200000 RMB□ |
| --- | --- |

1. Could this questionnaire represent the impact of vitiligo to you?

□Yes □Nearly □Not quite □No

1. Is there anything concerns vitiligo that we haven’t investigated?

1. Are you willing to participate in similar investigations in the future?

□Yes □No
